# Advances in extracellular vesicle (EV) biomarkers for precision diagnosis and therapeutic in colorectal cancer

**DOI:** 10.3389/fonc.2025.1581015

**Published:** 2025-07-16

**Authors:** Li Zhang, Hui Shen, Nenghua Zhang

**Affiliations:** ^1^ School of Life Sciences, Zhejiang Chinese Medical University, Hangzhou, Zhejiang, China; ^2^ Jiaxing Hospital of Traditional Chinese Medicine, Zhejiang Chinese Medical University, Jiaxing, Zhejiang, China

**Keywords:** colorectal cancer, extracellular vesicles, biomarkers, precision diagnosis, treatment

## Abstract

Colorectal cancer (CRC) is the second most common cause of cancer-related mortality worldwide and one of the most frequently diagnosed malignancies. Conventional CRC screening techniques—such as colonoscopy and pathologic biopsy—are invasive procedures that often cause patient discomfort and carry risks of complications. Recently, extracellular vesicles (EVs) have gained prominence as a promising area of investigation in oncology research. EVs are nanoscale, membrane-bound particles secreted by cells, which encapsulate and protect nucleic acids, proteins, and other biomolecules within their phospholipid bilayer structure. This protective characteristic renders EVs highly suitable as non-invasive diagnostic biomarkers for CRC, as well as efficient nanocarriers for targeted drug delivery vehicles. This review examines the functional roles, regulation mechanisms, and translational potential of EVs in CRC. Specifically, it investigates how EVs drive CRC pathogenesis through tumour microenvironment remodeling, immune suppression, and metastatic dissemination. Additionally, the review examines relevant methodologies for EV sample collection, isolation, and detection, which are critical for translating EV-based diagnostics and therapeutics into clinical practice. In conclusion, EVs represent a transformative approach in CRC research, offering non-invasive diagnostic tools and innovative therapeutic strategies. By integrating advanced methodologies and elucidating the regulatory mechanisms of EVs, this review provides valuable insights for advancing precision medicine in CRC, ultimately improving patient outcomes and reducing the global burden of this disease.

## 1 Introduction

### 1.1 Colorectal cancer epidemiology and therapeutic challenges

Colorectal cancer (CRC), a predominant gastrointestinal malignancy in the United States, ranks as the third most common cancer nationally in terms of both incidence and mortality when stratified by gender (1). In China, CRC is the second most frequently diagnosed cancer among men and the fourth leading cause of cancer-related deaths. Among women, it ranks fourth in incidence and second in mortality (2). Although the overall incidence of CRC has declined by 1.8% annually over the past decade (2012–2021), rates continues to rise among adults under 50 years of age. Notably, 10–12% of new cases are classified as early-onset CRC, often lacking identifiable risk factors (3). Current treatment modalities for CRC encompass surgical resection, radiotherapy, chemotherapy, targeted therapy, and immunotherapy. Standard chemotherapeutic regimens typically incorporate 5-fluorouracil and oxaliplatin, whereas targeted approaches employ monoclonal antibodies (e.g., cetuximab) and anti-VEGF agents (e.g., bevacizumab) (4, 5). Despite these therapeutic advances, the emergence of drug resistance frequently results in suboptimal survival outcomes and diminished quality of life for CRC patients.

### 1.2 Colorectal cancer pathogenesis: drivers and knowledge gaps

The pathogenesis of CRC is driven by genetic mutations, aberrant gene expression, and altered methylation patterns, which collectively dysregulate critical signalling pathways to promote tumour growth and metastasis. Key mutations in TP53 and KRAS, for example, are strongly associated with CRC metastasis and serve as important prognostic indicators (6). Nevertheless, the exact mechanisms through which these driver gene alterations facilitate CRC progression remain incompletely characterised.

### 1.3 Comparative analysis of colorectal cancer screening modalities: strengths and limitations of colonoscopy and fecal immunochemical test

Colonoscopy and the fecal immunochemical test (FIT) are currently the two most widely used methods for colorectal cancer screening worldwide (7).

According to clinical research data, colonoscopy, as the gold standard for CRC screening, is limited in clinical practice by its invasive nature (including anesthesia-related risks) and complication rates, with 16.4–36.18 cases of severe bleeding and 7.62–8.50 cases of intestinal perforation per 10,000 procedures, along with the associated burden of substantial healthcare costs.are invasive, expensive, and poorly suited for population-wide implementation (8).

The fecal immunochemical test (FIT) is a non-invasive, cost-effective, and convenient screening method with high public acceptance, providing a novel option for colorectal cancer (CRC) detection (9). Recent cost-effectiveness studies comparing three CRC screening approaches in Asian populations demonstrate that FIT exhibits the most favorable incremental cost-effectiveness ratio (ICER) at USD 108,176, compared to USD 133,485 for the M3 fecal biomarker panel and USD 159,586 for colonoscopy. However, FIT shows the lowest CRC prevention rate (4.5%), significantly lower than the 50.2% prevention rate of the M3 biomarker and 51.3% of colonoscopy. Significant differences in compliance rates are also observed: FIT has the lowest adherence rate (60%), while colonoscopy and the M3 biomarker achieve near-perfect compliance rates of 98.9% and 99%, respectively. Comprehensive evaluation integrating cost-effectiveness, prevention rates, and compliance metrics reveals that although colonoscopy excels in prevention efficacy and adherence, the M3 biomarker emerges as a more cost-effective strategy by balancing economic and clinical outcomes, positioning it as a promising alternative for CRC screening (10). This underscores the pressing need for non-invasive biomarkers that would permit early, cost-effective, and reliable CRC detection.

Liquid biopsy-based biomarkers-including extracellular DNA, circulating tumour cells, and extracellular vesicle (EV)-derived nucleic acids and proteins—represent promising alternatives for CRC diagnosis and monitoring (11–13). EVs have attracted particular interest owing to their remarkable stability, high abundance in bodily fluids, and capacity to encapsulate tumour-specific molecular cargo. This review focuses specifically on EV biomarkers in CRC, examining their potential to transform clinical practice through improved early detection, prognosis evaluation, and treatment monitoring—ultimately leading to enhanced patient outcomes and quality of life.

## 2 EVs introduction

### 2.1 Definition of EVs

In recent years, extracellular vesicles (EVs) have become a major focus of owing due to their considerable potential as both therapeutic targets and biomarkers in a wide range of diseases. Initially considered mere cellular waste disposal mechanisms, EVs are now understood to play a crucial role in intercellular communication. Their minimally invasive nature further enhances their suitability for applications in cancer screening and diagnostics. EVs are broadly classified into three subtypes based on their size and biogenesis: exosomes, microvesicles (MVs), and apoptotic bodies. These lipid bilayer-enclosed structures encapsulate a diverse molecular cargo, including DNA, mRNA, circular RNA (circRNA), microRNA (miRNA), long non-coding RNAs (lncRNAs), proteins, lipids, and metabolites. EVs are ubiquitously present in bodily fluids such as blood, urine, and cerebrospinal fluid, highlighting their diagnostic and therapeutic potential (14, 15). Among these, exosomes have been the most extensively studied, followed by microvesicles.

### 2.2 Classification of EVs

Exosomes, which range from 40 to 160 nm in diameter, are formed through the inward budding of multivesicular bodies (MVBs) within cells. Following their formation, MVBs fuse with the plasma membrane, releasing exosomes into the extracellular space via exocytosis. Microvesicles (MVs), by contrast, are larger vesicles (100–1000 nm) formed through the direct outward budding of the plasma membrane. These vesicles mediate intercellular communication either through fusing with recipient cell membranes or via phagocytosis, thereby delivering their molecular cargo to target cells. Apoptotic bodies, the largest EVs (100–5000 nm), are produced during programmed cell death (apoptosis) (16, 17). They contain specialised signalling molecules and cellular debris that facilitate the efficient clearance of apoptotic cells while helping to prevent inflammatory responses. An overview of three extracellular vesicle subtypes—exosomes, microvesicles, and apoptotic bodies—highlighting their key characteristics is provided in **Table 1**.

**Table 1:** Comparative analysis of three main extracellular vesicle subtypes: size, biogenetic origin, and functional characteristics.

Vesicles	Diameter Size(nm)	Biogenetic Origin	Functions
Exosomes	40~160	Endosomes	Cell communication, immunomodulation, and tumor development
Microvesicles	100~1000	Plasma membrane	Signalling and pathological mediation
Apoptotic bodies	100~5000	Plasma membrane	Cell death signalling and removal of harmful cells

### 2.3 Characteristics of the double-layer membrane of EVs

A defining characteristic of EVs is their phospholipid bilayer membrane, which protects their internal components (e.g., proteins, nucleic acids, and lipids) from enzymatic degradation in circulation, thereby preserving their structural integrity and biological activity. This remarkable stability not only enhances their biomarker potential but also renders them ideal candidates for drug delivery systems. In contrast to conventional serological markers, EVs can effectively encapsulate therapeutic agents, shielding them from degradation while facilitating cell-specific targeting (18). These unique properties establish EVs powerful tools for advancing precision medicine, particularly in oncological diagnostics and therapeutic applications.

### 2.4 Biogenesis pathways and biochemical composition of exosomes

Exosomes, a subtype of extracellular vesicles (EVs), are generated through a tightly regulated, cell-type-dependent biogenesis process responsive to environmental cues. While EV biogenesis and secretion mechanisms have been extensively reviewed elsewhere, the formation of exosomes specifically involves three sequential stages: biogenesis, trafficking, and release.

**Figure 1:**
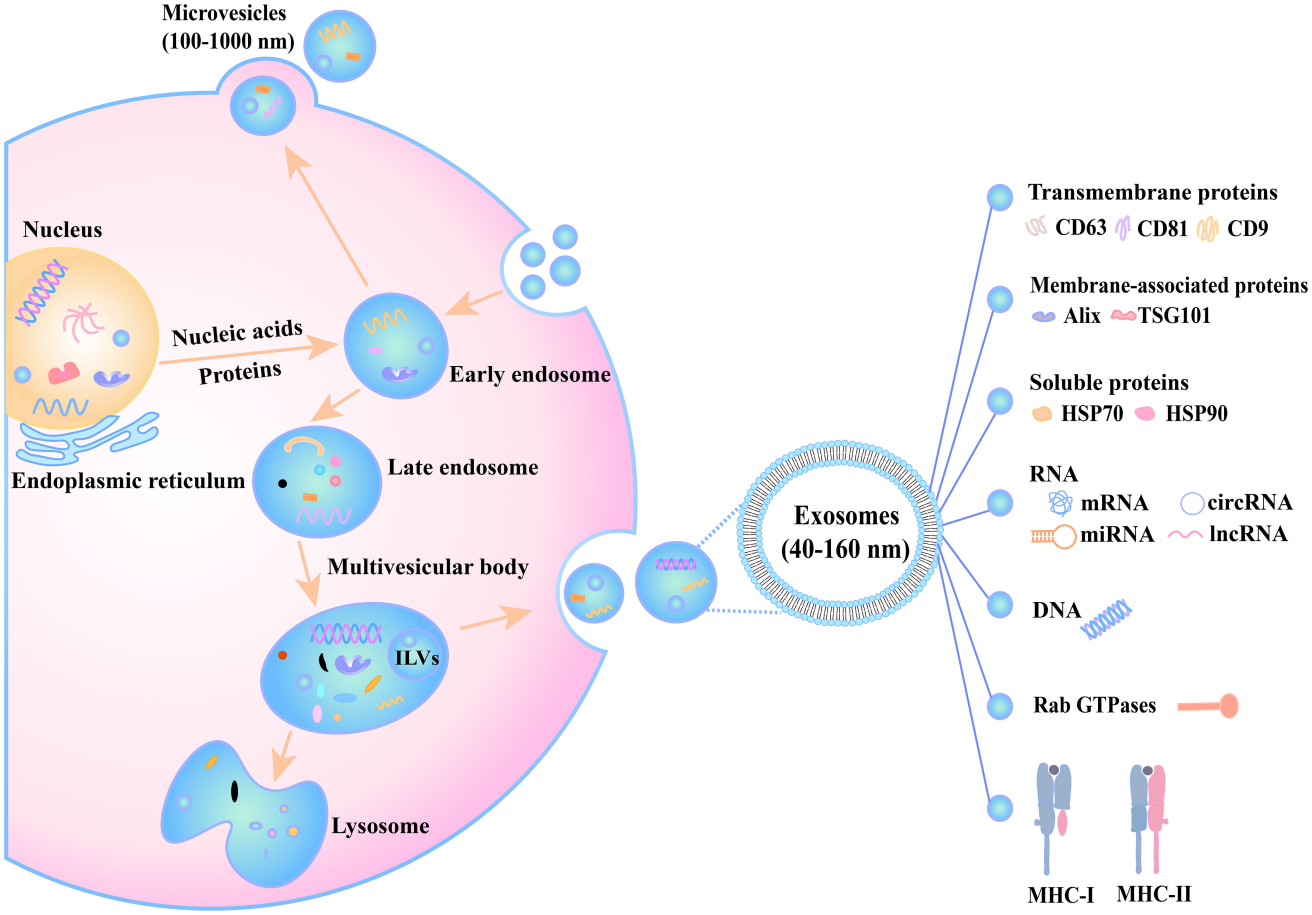
The biogenesis and secretion processes of extracellular vesicles. Inside the cell, nucleic acids and proteins originating from the nucleus and endoplasmic reticulum gradually participate in vesicle formation. The early endosome receives substances and develops into a late endosome, which then forms a multivesicular body. This multivesicular body has three potential fates: (1) fusion with a lysosome for content degradation, (2) interaction with an autophagosome, or (3) fusion with the cell membrane to release exosomes (40–160 nm) into the extracellular space. Additionally, the cell can release microvesicles (100–1000 nm) through plasma membrane budding. Exosome membranes are enriched with transmembrane proteins (CD63, CD81, CD9) and encapsulate diverse cargoes, including nucleic acids (miRNAs, circRNAs, lncRNAs), proteins (Alix, HSP70/90, and TSG101), metabolites, and immune regulators such as MHC-I and MHC-II, which mediate immune responses. These processes, regulated by factors such as Rab GTPases, play critical roles in intercellular communication and other physiological functions.

The process initiates when specific domains of the plasma membrane undergo endocytic internalization, giving rise to early endosomes. These compartments subsequently mature into multivesicular bodies (MVBs) through endosomal membrane invagination. Ultimately, MVBs fuse with the plasma membrane to release exosomes (19, 20). Notably, proteins (e.g., CD9), RNAs (such as miRNAs), and other biomolecules critically regulate each stage of exosome biogenesis and trafficking (20). The detailed processes of the biogenesis pathways and biochemical composition of exosomes are graphically presented in **Figure 1**.

### 2.5 Sources and isolation techniques for EVs

Currently, the isolation of extracellular vesicles (EVs) primarily relies on techniques tailored to their biochemical and physical properties. Ultracentrifugation remains the gold standard for EV separation, supplemented by alternative methods such as density gradient centrifugation, size-exclusion chromatography, immunoprecipitation, optical trapping, and magnetic bead-based isolation (21, 22). Additionally, commercial kits designed for exosome-specific (a subtype of EVs) isolation are widely employed. A summary of these methods and their applications across sample types (e.g., plasma, urine, and cerebrospinal fluid) is provided in **Table 2**. However, the field currently lacks a universally standardised protocol for EV isolation, owing primarily to the heterogeneous nature of EV subpopulations and the technical limitations of existing separation strategies. A major challenge involves minimising sample damage and contamination, issues that frequently arise due to the complex composition of biological matrices and the limitations of current isolation techniques. Consequently, there is a pressing need for innovative approaches to improve EV separation and purification, ensuring both reproducibility and high sample fidelity for downstream analyses like RNA sequencing or proteomics.

**Table 2:** Isolation techniques for extracellular vesicles from diverse biological samples.

EV sources	Isolation techniques	References
Serum	Ultracentrifugation, size exclusion chromatography, OptiPrep™ density gradient centrifugation	(23, 24)
Plasma	Ultracentrifugation^1^ ExoQuickTM precipitation solution (System Biosciences) Total Exosome Isolation Kit (Invitrogen) Nickel-based isolation (NBI)	(25)
Feces	Density gradient ultracentrifugation	(26, 27)
Urine	Ultracentrifugation^1^, chemical precipitation, immunoprecipitation	(28, 29)
Saliva	Norgen Saliva Exosome Purification Kit ExoQuick-TC ULTRA magnetic bead immunocapture assay	(30)
Cerebrospinal fluid	Differential centrifugation^1^ MagCapture Exosome Extraction Kit	(31, 32)
Breast milk (human)	Ultracentrifugation, density gradient centrifugation, commercial precipitation kit, size exclusion chromatography	(33)
Seminal plasma	Ultracentrifugation and size exclusion chromatography	(34)
Bile	Ultracentrifugation	(35)
Colonic luminal fluid aspirates	Ultracentrifugation	(36)

Various biological samples, including serum, plasma, feces, urine, saliva, cerebrospinal fluid, human breast milk, seminal plasma, bile, and colonic luminal fluid aspirate. This table summarizes representative methods and is not exhaustive. ^1^Common separation methods.

## 3 The relationship between EVs and the intestinal microenvironment

### 3.1 The intestinal microenvironment

The intestinal microenvironment (IME) constitutes a complex biological and chemical ecosystem comprising intestinal microbiota, epithelial cells, immune cells, and secretory components. Among these components, the gut microbiota contributes to numerous protective, structural, and metabolic functions within the intestinal epithelium. It plays a crucial role in maintaining a balanced microenvironment that regulates inflammatory responses and preserves intestinal homeostasis. Disruption of this delicate equilibrium has been implicated in the pathogenesis of inflammatory bowel disease (IBD), colorectal cancer (CC), and various systemic disorders (37). Recent research has prioritised therapeutic approaches to restore IME homeostasis in IBD patients, with particular emphasis on bioengineered probiotics. These advanced microbial therapeutics are specifically designed to simultaneously modulate: (1) gut microbiota composition, (2) host immune responses, and (3) cellular redox balance, thereby representing a novel and promising treatment strategy (38).

Furthermore, dysregulation of the gut microenvironment is increasingly recognised as a contributor to systemic inflammation and extra-intestinal pathologies, including myocardial injury. Such disturbances may disrupt intestinal redox homeostasis and compromise epithelial barrier integrity, thereby promoting immune dysfunction and exacerbating systemic inflammatory cascades (39). Recent studies have demonstrated that faecal/intestinal microbiota transplantation (FMT/IMT)-the transfer of minimally processed faeces into a recipient’s gastrointestinal tract-can restore gut microbiota homeostasis and ameliorate disorders linked to microbial dysbiosis. As a potential therapy for recurrent Clostridioides difficile infection (rCDI), FMT/IMT has achieved a success rate of approximately 90% alongside improved overall patient survival (40). However, controversies persist regarding certain aspects, such as the optimal timing of administration, and standardised protocols remain to be established (41). Collectively, these findings highlight the pivotal role of the IME in systemic health and disease, offering novel insights into the therapeutic potential of restoring intestinal homeostasis to mitigate systemic inflammation and immune dysregulation. Future research should focus on developing precision-based strategies to translate these mechanisms into clinical applications.

### 3.2 miRNAs derived from EVs regulate the intestinal microenvironment

A growing body of evidence highlights the crucial role of extracellular vesicles (EVs) in facilitating intercellular communication and maintaining intestinal homeostasis (42). Recent studies demonstrate that intestinal bacteria—including commensal, probiotic, and pathogenic strains—modulate the intestinal microenvironment (IME) and host health through bacterial extracellular vesicles (BEVs) (43). Furthermore, gut microbiota routinely produce outer membrane vesicles (OMVs), nanoscale vesicular structures originating from Gram-negative bacteria. Notably, BEVs and OMVs share remarkable similarities with mammalian exosomes in terms of size distribution, genetic content, and protein-lipid compositions (44, 45). These observations collectively emphasise the regulatory importance of BEVs, OMVs, and exosomes in maintaining intestinal equilibrium.

Emerging research indicates that exosomal microRNAs derived from host cells can modulate gut microbial dynamics, providing novel diagnostic and prognostic biomarkers, as well as potential therapeutic targets for gut-related disorders (46). Probiotics, recognised for their beneficial role in intestinal health, mediate their effects by regulating mucosal immunity, maintaining microbial balance, and improving nutrient absorption (47). Notably, Clostridium butyricum has demonstrated protective properties in various models of intestinal inflammation. Extracellular vesicles derived from Clostridium butyricum (CbEVs) have been found to inhibit pro-inflammatory signalling pathways, including nuclear factor kappa-light-chain-enhancer of activated B cells (NF-κB) and mitogen-activated protein kinase (MAPK), while upregulating miR-199a-3p expression via interactions with MAP3K4 (48). These findings suggest that CbEVs and miR-199a-3p could serve as promising therapeutic agents or targets for inflammatory bowel disease (IBD).

Beyond bacterial exosomes, plant-derived exosomal miRNAs and herbal exosomes have also exhibited anti-inflammatory properties in the gut. Dendrobium officinale polysaccharides (DOP), extracted from the medicinal plant Dendrobium officinale, exhibit multiple therapeutic effects including antioxidant, immunomodulatory, and gastrointestinal protective activities (49). Studies indicate that DOP enhances the release of miR-433-3p in small extracellular vesicles (sEVs), which may attenuate intestinal inflammatory responses (50). Furthermore, research in murine models has demonstrated that total body irradiation (TBI) induces intestinal mucosal barrier damage, microbial dysbiosis, and destabilisation of the gut microenvironment. Notably, exosomal miRNA-142-5p—upregulated in donor mice—significantly reduced TBI-induced gastrointestinal toxicity and tissue damage (51). These findings suggest a potential therapeutic strategy for mitigating radiation-induced gastrointestinal injury in exposed individuals.

In summary, these findings highlight the multifaceted roles of EVs and EV-derived miRNAs in maintaining intestinal microenvironmental homeostasis, primarily achieved through modulation of signaling pathways, inflammatory responses, and immune activity. An overview of EV-derived miRNA functions in both homeostasis maintenance and pathological regulation is presented in **Table 3**. Building on these insights, future research should prioritize exploring the broader therapeutic potential of EVs, including their diverse molecular cargo and regulatory networks, to develop targeted therapies for gut-related pathologies and systemic inflammatory disorders.

**Table 3:** Functions of EV-derived miRNAs in intestinal homeostasis and pathological regulation.

Types	Expression	Functions	References
miR-199a-3p	Up	Improves intestinal barrier integrity and inhibits inflammatory responses in mice with colitis;	(48)
miR-433-3p	Up	Prevents accumulation of inflammatory factors induced by intestinal macrophage hyperactivity and restores intestinal microenvironmental homeostasis;	(50)
miR-142-5p	Up	Enhanced protection against radiation enteritis;	(51)
miR-181b-5p	Up	Promoting the polarization of M2 macrophages in the intestine, secreting anti-inflammatory factors, and alleviating intestinal inflammatory conditions;	(52)
miR-200b-3p	Up	Regulates the composition of the flora, increases the abundance of probiotics in the inflammatory flora, and improves the flora structure;	(52)

These functions include maintaining barrier integrity, anti-inflammation, protection against radiation-induced enteritis, M2 macrophage polarization, immune regulation, and intestinal microbiota modulation. Specific miRNAs are shown in the table.

## 4 The role of new EV markers in colorectal cancer

### 4.1 The regulatory role and clinical application potential of EV-derived miRNAs in the progression of colorectal cancer

#### 4.1.1 miRNA

MicroRNAs (miRNAs), a class of non-coding single-stranded RNAs—approximately 22–25 nucleotides in length—are of particular interest due to their pivotal role in post-transcriptional gene regulation. miRNAs function by binding to complementary sequences on target messenger RNAs (mRNAs), thereby modulating protein synthesis through either mRNA degradation or translational repression (53, 54).

#### 4.1.2 miRNAs derived from EVs as potential biomarkers for colorectal cancer

In colorectal cancer (CRC), exosomal miRNAs have emerged as promising biomarkers due to their dysregulated expression during tumour progression (55). For instance, elevated levels of miR-17-5p, miR-181a-5p, miR-18a-5p, and miR-18b-5p are observed in plasma exosomes from colorectal cancer (CRC) patients, suggesting their potential as non-invasive diagnostic biomarkers (56). Additionally, reduced exosomal miR-150 expression has been associated with CRC liver metastases and correlated with significantly poorer overall survival (OS) time (33.3 months versus 43.3 months, P = 0.002), underscoring its prognostic value. Mechanistically, decreased miR-150 expression enhance the survival and invasive potential of CRC cells, further supporting its dual role as a prognostic marker and therapeutic target (57). Moreover, another study has found that specific exosomal miRNAs—including miR-19b, miR-21, miR-222, and miR-92a—demonstrate both diagnostic and prognostic value in early-stage CRC. Notably, miR-21 and miR-19b demonstrate superior diagnostic performance compared to the conventional biomarker carcinoembryonic antigen (CEA). miR-21 achieves an area under the curve (AUC) of 0.981, while miR-19b attains an AUC of 0.951—both significantly outperforming CEA (AUC = 0.906) in terms of sensitivity and specificity (58). Furthermore, EV-associated miRNAs such as miR-210, which shows elevated expression in CRC cells, correlate with patient survival outcomes. Mechanistic studies reveal that miR-210 modulates the XIST/NME1 pathway, thereby suppressing CRC tumorigenesis (59).

#### 4.1.3 Possible regulatory roles of EV-derived miRNAs in the progression of colorectal cancer

Small extracellular vesicles (sEVs), a subset of EVs ranging from 30 to 150 nm in size, exhibit superior diagnostic utility compared to free plasma miRNAs due to their enhanced stability against RNase A degradation. This characteristic renders sEVs particularly valuable for the early detection of colon cancer (60). Furthermore, EV-derived miRNAs have been demonstrated to modulate key tumour characteristics, including proliferation, invasion and metastasis, through regulating of target genes and signalling pathways. For instance, miR-224-5p overexpression in EVs derived from colorectal cancer-associated fibroblasts (CAFs) promotes CRC cell proliferation, migration and apoptosis resistance by targeting the sodium bicarbonate cotransporter 1 (SLC4A4) (61). Conversely, reduced levels of miR-193a-5p in plasma EVs from CRC patients suppress migration and invasion through regulating of cut-like homeobox 1 (CUX1) and intersectin 1 (ITSN1) (62). These findings demonstrate the dual regulatory roles of EV-derived miRNAs in CRC, which may function either as oncogenic drivers or tumour suppressors depending on their specific targets and cellular context. Their capacity to modulate tumour behaviour through complex molecular networks underscores their potential as diagnostic biomarkers, prognostic indicators, and therapeutic targets). Based on these findings, to integrate existing research and clarify the functional characteristics and mechanistic details of each miRNA, key information on relevant miRNAs is organized below (**Table 4**).

**Table 4:** Overview of EV-derived miRNAs related to colorectal cancer: expression patterns, detection methods, sources, functions, and mechanisms.

miRNAs	Expression	Methods	Sources	Functions	Mechanisms	References
miR-224-5p	Up	qRT-PCR	HCT-116/SW-480/Caco-2/LoVo/T84/NCM-460/CAFs cells	Proliferation/migration/invasion/apoptosis	Targets SLC4A4 gene	(61)
miR-193a-5p	Down	qRT-PCR	HCT-8/SW-480/CCC-HIE-2 cells/plasma (human)	Proliferation/migration/invasion	Targets CUX1 and ITSN1 genes	(62)
miR-25	Up	qRT-PCR	HCT-116/SW-480/SW-620/LOVO/NCM-460 cell	proliferation/migration/invasion	Inhibits SIRT6 and mediates Lin28b/NRP-1 axis	(63)
miR-27b-3p	Up	qRT-PCR	DLD-1/SW-620/HT-29/HCT-116/SW-480/NCM-460 cells	Metastasis/vascular permeability	Targets STAT3/hnRNPA1/miR-27b-3p signaling cascade	(64)
miR-221-3p	Up	qRT-PCR	HCT-116/Caco-2 cells/serum (human)	Metastasis/angiogenesis	Targets SOCS3 regulating STAT3/VEGFR-2 axis	(65)
miRNA-222	Up	qRT-PCR	SW-480/HCT-116/293T/NCM-460/MSC cells	Invasion/immune escape	Targets ATF3, inhibit AKT1 transcription, mediate AKT pathway	(66)
miR-183-5p	Up	qRT-PCR	DLD-1/HT-29/H CT-116/NCI-H508/HMEC-1 cells/nude mouse serum	Proliferation/migration/angiogenesis	Targets FOXO1 gene	(67)
miR-34a-5p	Down	qRT-PCR	HCT-116/SW-480/LoVo/HEK-293 cells	Proliferation/migration/invasion/apoptosis	Targets miR-34a-5p/c-MYC/DNMT3a/PTEN axis	(68)
miR-200	Up	qRT-PCR	DLD-1/HCT-116/SW-620/SW-480 cells	Differentiation	Targets miR-200/ZEB1 axis	(69)
miR-106b-3p	Up	qRT-PCR	HCT-116/SW480/SNU-C1/SW-1116/LoVo/KM12SM/NCM-460 cells/serum (human)	Metastasis	Targets DLC-1	(70)
miR-203a-3p	Up	qRT-PCR	Plasma (human)	Metastasis	Targets PTEN/AKT/PI3K and CXCL12/CXCR4/NF-κB pathway	(71)
miR-25-3p/miR-130b-3p/miR-425-5p	Up	qRT-PCR	HCT-116/SW-620/RAW264.7/THP-1 cells	Metastasis	Activates CXCL12/CXCR4 axis	(72)
miR-15a	Up	qRT-PCR	LoVo/HCT-116/adMSCs cells	Proliferation/migration/invasion/metastasis/apoptosis	Targets KDM4B/HOXC4/PD-L1 axis	(73)
miR-146a-5p	Up	qRT-PCR	serum (human)	Metastasis	Activates CXCL12/CXCR7 axis	(74)
miR-155-5p	Up	qRT-PCR	serum (human)	Transfer	Activates CXCL12/CXCR7 axis	(74)
miR-181a-5p	Up	qPCR	serum (human)	Metastasis	Targets SOCS3 activates IL-6/STAT3, leading to CCL20/CCR6/ERK1/2/Elk-1/miR-181a-5p feedback loop	(75)
miR-361-3p	Up	qRT-PCR	HCT-116/HT-29 cells/serum (human)	Proliferation	Targets TRAF3 to activate the non-canonical NF-κB pathway	(76)
let-7a miRNA	Up	qPCR	SW-48/SW-480/HT-29/SW-620/FHC cells/serum (human)	Proliferation/migration/invasion	Targets let-7a-SNAP23 axis	(77)
miR-200c-3p	Down	qPCR	Serum (human)	Migration/invasion/apoptosis	Targets ZEB-1	(78)

These data provide valuable reference for subsequent research and clinical applications in colorectal cancer.

### 4.2 The regulatory role and clinical application potential of EV-derived circRNAs in the progression of colorectal cancer4.2.1 Circular RNA

Circular RNAs (circRNAs) constitute a class of endogenous RNAs produced through back-splicing events, forming covalently closed-loop structure devoid of 5’ and 3’ termini. This distinctive configuration confers exceptional stability, making circRNAs resistant to exonuclease-mediated degradation relative to their linear counterparts (79). Growing evidence indicates that dysregulated circRNA expression in colorectal cancer (CRC) cells facilitates tumour progression by suppressing tumour suppressor genes, thereby promoting tumour initiation, invasion, and metastasis (80). Moreover, circRNAs encapsulated within extracellular vesicles (EVs) are emerging as valuable biomarkers for the diagnosis, prognosis, and therapeutic monitoring of gastrointestinal cancers (81).

#### 4.2.2 CircRNAs derived from EVs as potential biomarkers for colorectal cancer

For example, hsa_circ_0003270 (circGAPVD1)—a circRNA involved in the circGAPVD1-miRNA-mRNA regulatory network - shows significant upregulation in plasma EVs from CRC patients and correlates with TNM staging, demonstrating its potential as both a diagnostic and prognostic marker (82). CircRNAs function as competitive endogenous RNAs (ceRNAs) or “miRNA sponges” by containing conserved miRNA binding sites that sequester miRNAs and thereby regulate downstream target gene expression. A prominent example is circPACRGL, which is abundant in CRC cell-derived exosomes. This circPACRGL sponges miR-142-3p and miR-506-3p, resulting in increased transforming growth factor-β1 (TGF-β1) expression. This process enhances CRC cell proliferation, migration and invasion, while simultaneously inducing neutrophil polarisation from an anti-tumour (N1) to a pro-tumour (N2) phenotype (83). These findings highlight the multifaceted roles of EV-derived circRNAs in CRC pathogenesis and their promise as robust biomarkers for early detection, risk stratification and therapeutic targeting. Through miRNA sponging and other regulatory mechanisms, circRNAs emerge as pivotal regulators of gene expression networks in cancer biology. To clearly illustrate the intrinsic connections among these research findings, this article summarizes EV-derived circRNAs in CRC cell lines and clinical samples in terms of expression patterns, regulatory mechanisms, and other aspects in **Table 5**.

**Table 5:** Summarizes data on six upregulated and one downregulated EV-derived circRNAs, including their detection methods, biological sources, functions in CRC progression, and associated regulatory mechanisms.

circRNAs	Expression	Methods	Sources	Functions	Mechanisms	References
hsa_circ_0003270(circGAPVD1)	Up	qRT-qPCR	Plasma (human)	TNM staging/metastasis	circGAPVD1-miRNA-mRNA regulatory network	(82)
circPACRGL	Up	qRT-PCR	HCT-116/SW-480 cells	Proliferation/migration/invasion/differentiation	Targets miR-142-3p/miR-506-3p-TGF-β1 axis	(83)
circ-133	Up	RT-qPCR	HCT-116/SW-480 cells/plasma (human)	Migration	Targets GEF-H1/RhoA axis	(84)
circ_0005615	Up	qRT-PCR	serum (human)	Proliferation/migration/invasion/apoptosis	Targets miR-873-5p/FOSL2 pathway	(85)
circ_CCDC66	Up	qPCR	THP-1/HEK-293T/CD8+T/SW-480/HCT-116/FHC cells	Immune escape/growth/migration	circRNA CCDC66/microRNA-342-3p/MTDH axis	(86)
hsa_circ_0001739	Up	qRT-PCR	SW-480/SW-620/HCT-8/HIEC cells	metastasis	Regulates miR-218-5p/FTO-m6A/MYC axis	(87)
circLPAR1	Down	qRT-PCR	Plasma (human)	Proliferation/migration/invasion	Regulates circLPAR1/METTL3-eIF3h/BRD4	(88)

These circRNAs play critical roles in the initiation and development of colorectal cancer.

### 4.3 The regulatory role and clinical application potential of EV-derived lncRNAs in the progression of colorectal cancer

#### 4.3.1 Long non-coding RNA

Long non-coding RNAs (lncRNAs) represent a class of RNA molecules exceeding 200 nucleotides in length that lack protein-coding capacity due to the absence of open reading frames. Substantial evidence has established that lncRNAs are frequently dysregulated in multiple malignancies, including colorectal cancer (CRC), where they play pivotal roles in tumorigenesis, progression, and metastasis (89). Growing research indicates these molecules show considerable potential as biomarkers for CRC diagnosis, prognosis and treatment response assessment.

#### 4.3.2 LncRNAs derived from EVs as potential biomarkers for colorectal cancer

For example, the lncRNA HOTAIR (HOX transcript antisense RNA) contributes significantly to CRC progression, demonstrating an inverse correlation between its elevated expression levels and both overall survival (OS) and relapse-free survival (RFS) in CRC patients (90). This relationship establishes HOTAIR as a promising prognostic indicator, where high expression levels predict unfavourable clinical outcomes. Furthermore, lncRNAs actively modulate the tumour microenvironment to influence CRC progression and metastasis. A particularly significant example is the lncRNA CRNDE-h, which shows marked upregulated in serum exosomes from CRC patients and exhibits strong associations with both regional lymph node involvement and distant metastases. Demonstrating a sensitivity of 70.3%, specificity of 94.4%, and an area under the ROC curve of 0.892, CRNDE-h surpasses conventional biomarkers including carcinoembryonic antigen (CEA) (AUC: 0.688; sensitivity: 37.16%; specificity: 88.75%) in diagnostic performance (91).

In contrast, several lncRNAs demonstrate reduced expression in CRC, providing additional prognostic value. Specifically, the lncRNAs H19, HOTTIP and HULC show significant downregulation in serum exosomes from CRC patients. Of particular note, diminished HOTTIP expression in serum-derived exosomes has emerged as a potential predictive biomarker for CRC, further highlighting the diagnostic and prognostic utility of lncRNAs (92).

Based on the above studies, this article summarizes the expression patterns, regulatory mechanisms, and other aspects of different EV-derived lncRNAs in colorectal cancer progression, as shown in **Table 6**. These observations underscore the dual regulatory function of lncRNAs in CRC, which can act as either oncogenic drivers or tumour suppressors, with their expression patterns offering clinically relevant insights into tumour behaviour and patient outcomes.

**Table 6:** Shows the expression patterns, detection method, biological sources, functions, and molecular mechanisms of EV-derived lncRNAs in colorectal cancer cell lines and clinical samples.

lncRNAs	Expression	Method	Sources	Functions	Mechanism	References
lncRNA AC159540.1	Up	qRT-PCR	SW-480/SW-620/HCT-8/HIEC cells	Metastasis	Regulates miR-218-5p/FTO-m6A/MYC axis	(87)
lncRNA SNHG3	Up	qRT-PCR	CAFs/NFs/HCT-116 cells	Proliferation	Targets miR-34b-5p/HuR/HOXC6 axis	(93)
lncRNA 91H	Up	qRT-PCR	HCT-8/HCT-116/FHC cells/serum (human)	Migration/invasion	Modulates HNRNPK expression	(94)
lncRNA HOTTIP	Up	qRT-PCR	HCT-116/SW-620/FHC/LoVo/HT-29/SW-480/SW-1116/Caco-2 cells/plasma (human)	Drug-resistant	Regulates HOTTIP/miR-214/KPNA3 network	(95)
lncRNA UCA1	Down	qRT-PCR	HCT-116/Caco-2 cells/serum (human)	Metastasis	Regulates ceRNA network	(96)

### 4.4 The regulatory role and clinical application potential of EV-derived Proteins in the progression of colorectal cancer

#### 4.4.1 Proteins

Extracellular vesicles (EVs) contain a diverse repertoire of proteins, many of which play significant roles in oncogenesis and tumour progression. Prominent examples include heat shock protein 90 (HSP90), annexin A1/2 (ANXA1/2), and lactate dehydrogenase A (LDHA), proteins that demonstrate characteristic dysregulation or mutational profiles across multiple malignancies (97). Specifically in colorectal cancer (CRC), the proteins cargo of small extracellular vesicles (sEVs) has shown particular promise as biomarkers for both early detection and disease monitoring (98).

#### 4.4.2 Proteins derived from EVs as potential biomarkers for colorectal cancer

A landmark proteomic study identified six CRC-specific sEV proteins that effectively discriminate between early-stage CRC, advanced CRC, and healthy controls: glutamate-cysteine ligase modulatory subunit (GCLM), Kell blood group complex subunit (KEL), apolipoprotein F (APOF), complement factor B (CFB), phosphodiesterase 5A (PDE5A), and 5’-aminoimidazole-4-carboxamide ribonucleotide formyltransferase/IMP cyclohydrolase (ATIC) (99). Furthermore, membrane-bundle proteins family members, which show significant enrichment in EVs, exhibit strong correlations with CRC prognosis, reinforcing their dual diagnostic and prognostic value (100, 101). Notably, four annexin family members (e.g., A3, A4, A5, and A11) demonstrate significantly higher sensitivity (> 75%) than the conventional biomarker CEA (sensitivity: 38.8%) in early colorectal cancer (CRC) detection, highlighting their potential as novel non-invasive diagnostic tools for clinical applications (102).

Non-invasive screening approaches, particularly faecal- and blood-based assays, represent the current gold standard for CRC detection (103). Of particular significance, fecal-derived EVs (fEVs) from CRC patients elevated expression of specific proteins biomarkers, including the transmembrane glycoproteins A33 (also known as GPA33) and CD147 (basigin). ROC analysis revealed that both CD147 (AUC = 0.903) and A33 (AUC = 0.904) effectively discriminated CRC patients from healthy controls. The combined assessment of CD147 and A33 enhanced diagnostic performance, yielding an improved AUC of 0.913. Notably, CD147, A33, and their combination each demonstrated 89% clinical sensitivity, substantially outperforming carcinoembryonic antigen (CEA; sensitivity = 40%). These findings establish fEV proteins as highly promising non-invasive biomarkers for CRC diagnosis and prognosis (104). However, implementation challenges persist, including logistical and technical hurdles in faecal sample collection/processing, along with the substantial costs associated with large-scale sample handling and transportation (105).

The protein components of extracellular vesicles demonstrate unique potential as novel biomarkers for precision medicine in CRC. To further elucidate their mechanisms and clinical applications, this review synthesizes recent data on EV-derived proteins associated with CRC pathological progression (see **Table 7**), providing valuable references for early diagnosis and mechanistic exploration.

**Table 7:** Summarizes the characteristics of EV-derived proteins closely associated with the pathological progression of colorectal cancer, covering four aspects: 1) expression patterns; 2) detection methods; 3) biological sources, and 4) functional roles.

Proteins	Expression	Methods	Sources	Functions	References
CD147/A33	Up	ELISA/TMT-LC-MS	Feces (human)	Diagnostic/Prognostic	(104)
CD59	Up	AIMS/LC-MS/MS	HT-29/SW-480/Colo-205/SW-620 cells/Plasma (human)	Metastasis	(106)
Tetraspanin 9	Up	AIMS/LC-MS/MS	HT-29/SW-480/Colo-205/SW-620 cells/Plasma (human)	Metastasis/TNM staging	(106)
SPARC	Up	ELISA/MS/MS	SW-480/HCT-116 cells/Plasma (human)	Metastasis	(107)
LRG1	Up	ELISA/TMT-LC-MS	SW-480/HCT-116 cells/Serum (human)	Metastasis	(107)
CPNE3	Up	ELISA	Plasma (human)	Diagnostic/Prognostic	(108)
CAPS1	Up	LC-MS	HT-29/SW-480/FHC/293T cells	Migration	(109)
S100A9	Up	ELISA	CT-26/SW-480 cells/Plasma (human, mouse)	Regeneration	(110)

### 4.5 Common mutant genes in colorectal cancer are involved in the secretion regulation, composition, and metastasis-mediated biological effects of extracellular vesicles

Accumulating evidence highlights the pivotal role of genetic mutations in colorectal cancer (CRC) pathogenesis, where alterations in key oncogenes and tumour suppressor genes driving tumour initiation, progression and metastasis (111). Six principal driver genes—adenomatous polyposis coli (APC), v-Raf murine sarcoma viral oncogene homolog B1 (BRAF), phosphoinositide-4,5-bisphosphate 3-kinase catalytic subunit alpha (PIK3CA), SMAD family member 4 (SMAD4), KRAS, and TP53—represent potential biomarkers for CRC metastasis and therapeutic targets (112). Notably, KRAS emerges as one of the most commonly mutated oncogenes in CRC, with mutations detected approximately 40% of cases, underscoring its fundamental role in CRC biology (113).

Recent studies have demonstrated that exosomes carrying KRAS mutations play a pivotal role in promoting tumour progression by facilitating neutrophil aggregation and the formation of neutrophil extracellular traps (NETs). These exosomes transfer mutant KRAS to recipient cells, resulting in elevated interleukin-8 (IL-8) levels, which subsequently drive neutrophil recruitment and NET formation. This cascade of events accelerates the metastatic spread of CRC and exacerbates disease severity (114). In addition to KRAS, recent studies have also revealed the critical functions of APC and transforming growth factor β receptor 2 (TGFBR2, a key component of the TGF-β signaling pathway) in regulating the properties of EVs and indicated that TP53 mutations are associated with EV-mediated biological effects in the tumor microenvironment (**Table 8**). These findings highlight the profound highlight of exosomal genetic material on cancer biology and its ability to remodel the tumour microenvironment. Exosomes mediate intercellular communication and transfer oncogenic cargo, such as mutant KRAS, underscoring their potential as both biomarkers and therapeutic targets in CRC. By modulating immune responses and promoting metastatic niches, exosomes contribute to the aggressive behaviour of CRC, presenting new opportunities for therapeutic intervention.

**Table 8:** Shows that in colorectal cancer: 1) mutant APC regulates extracellular vesicle (EV) secretion, while mutant TGFBR2 modulates EV composition; EV-mediated transfer of mutant KRAS and TP53 drives functional impacts on tumor progression.

Genes	Extracellular Vesicle Sources	Functional Impacts	References
KRAS	DKs-8/DKO-1 cells/APC-WT/APC-KRAS^G12D^ type mouse serum	IL-8 activation/neutrophil recruitment/NETs formation	(114)
Apc	HCT-116/SW-620/HT-29/SW-1222/ATCC-1459 cells/Human organoids	Hypoxia activates Wnt, boosts EV secretion, and enhances organoid colony formation	(115)
TGFBR2	HCT-116 cell	Altered miRNA profile of EVs and parental MSI CRC cells	(116)
TP53	HCT-116/HT-29/CCD-18Co/WI-38 cells	Promotion of fibroblast proliferation and CRC growth	(117)

This table covers EV biological sources.

### 4.6 DNA derived from EVs as potential biomarkers for colorectal cancer

Tumour-derived exosomes contain double-stranded DNA (dsDNA), which serves as a potential biomarker by reflecting the mutational profile of the originating cancer cells (118). In colorectal cancer (CRC), circulating tumour DNA (ctDNA), predominantly released from tumour tissues, has emerged as a powerful tool for precision medicine, enabling the detection of tumour-specific mutations and guiding therapeutic decisions (119). Comparative analyses of DNA from ctDNA and small extracellular vesicle DNA (sEV DNA) in patients with metastatic colorectal cancer (mCRC) have revealed concordant mutations in key driver genes such as KRAS and BRAF. Notably, DNase I treatment significantly reduced sEV DNA levels, with only 10% of the original DNA remaining, underscoring the potential of sEV DNA as a reliable source for identifying KRAS and BRAF mutations in mCRC (120). Furthermore, extracellular vesicle DNA (evDNA) has demonstrated a 6.67% higher sensitivity than circulating free DNA (cfDNA) in detecting KRAS G12D and G13D mutations in the plasma of CRC patients. This enhanced sensitivity suggests that evDNA could complement existing methodologies for mutation analysis and treatment monitoring in CRC, offering a more comprehensive approach to assessing tumour dynamics (121).

Furthermore, a study analysing plasma samples from mCRC and CRC patients demonstrated a positive correlation between disease progression and plasma exosome levels. The copy number and mutation abundance score of KRAS in exosomal DNA were significantly elevated compared to healthy controls. Following primary tumour resection, a marked reduction was observed in both the copy number and mutation abundance score of KRAS G12V/D variants within metastatic lesions. Notably, plasma exosomal wild-type and mutant KRAS median copy numbers (125/ml and 37/ml respectively) were identified as predictive biomarkers for OS. These findings underscore the clinical utility of plasma exosome quantification and their genetic cargo in the prognostic assessment of mCRC (122).

Although the above research achievements have brought hope for the clinical application of EV-DNA, its isolation and extraction remain a crucial challenge in practical implementation. Current methods, including commercial kits, face limitations such as low yield, contamination susceptibility, compromised DNA integrity, lack of standardized methods, and methodological variability (123, 124). To mitigate surface-associated DNA contamination, most studies employ pre-treatments with nucleases (e.g., DNase I, Exonuclease I) (125). These technical bottlenecks restrict both the advancement of EV-DNA research and its clinical translation, necessitating innovative solutions.

Despite these challenges, emerging strategies demonstrate promise. The integration of ctDNA and sEV DNA analyses offers a powerful approach for characterising the mutational landscape of CRC, significantly improving both diagnostic precision and prognostic evaluation. These liquid biopsy techniques facilitate: non-invasive monitoring of tumour evolution; early identification of resistance mechanisms; real-time assessment of therapeutic response. By combining the complementary combining of ctDNA and evDNA, clinicians can: gain deeper insights into tumour heterogeneity; develop more personalised treatment strategies; ultimately enhance patient outcomes.

**Figure 2:**
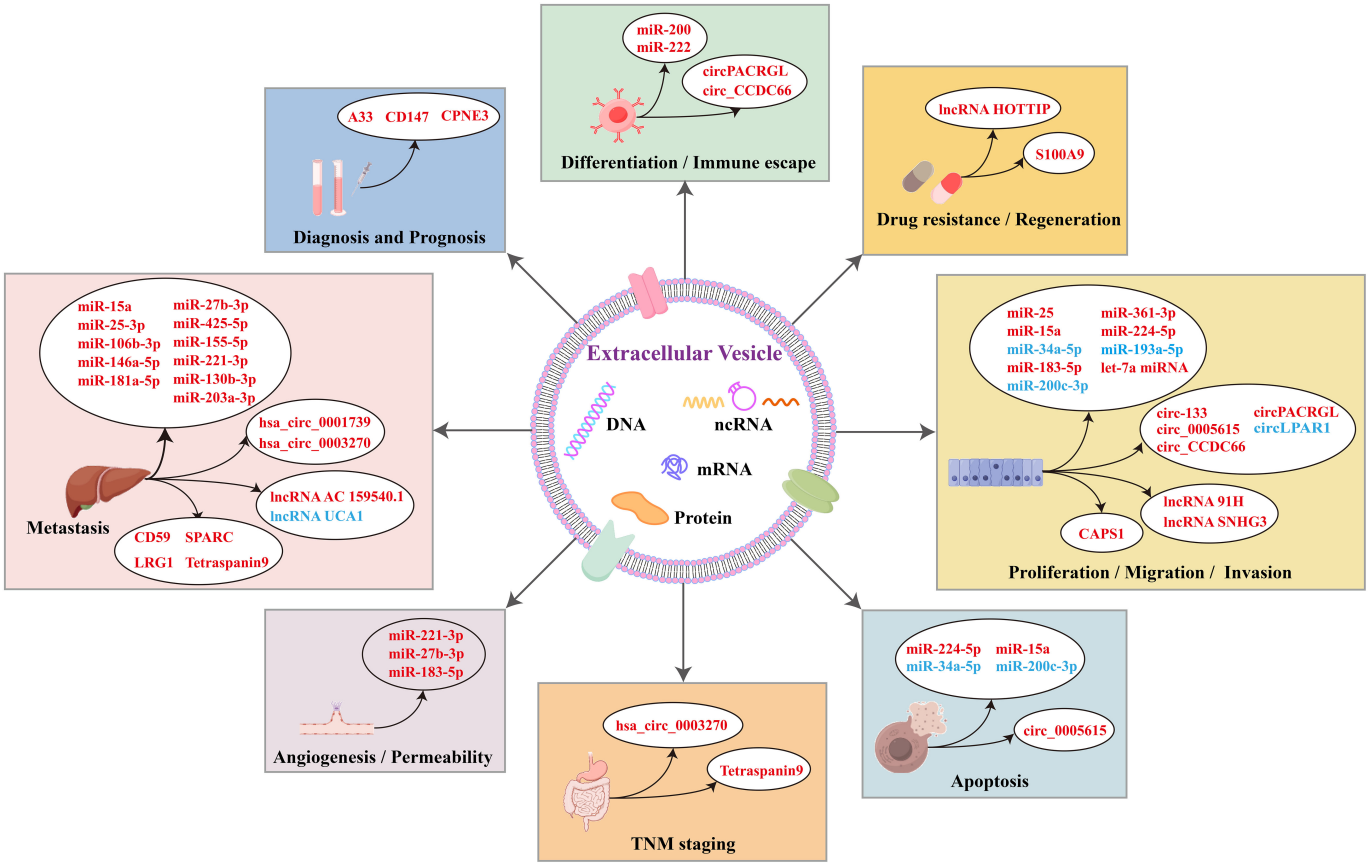
Mechanisms of biological functions related to EV-derived components, including miRNAs, circRNAs, lncRNAs, and proteins in colorectal cancer. The figure covers eight major aspects of their functions, including: 1) involvement in diagnosis and prognosis; 2) regulation of differentiation and immune escape; 3) induction of drug resistance and regeneration; 4) promotion of proliferation, migration, and invasion; 5) modulation of apoptosis; 6) TNM staging; 7) angiogenesis and permeability; and 8) metastasis and therapy. These EV-derived components can further interact through various molecular mechanisms, thereby influencing the biological processes of CRC cells. These insights provide valuable references for targeting tumor cell-related biological behaviors in CRC treatment. In each box, the font color legend is reported as follows: red represents upregulated genes, and blue represents downregulated genes.

Extracellular vesicles (EVs) have established themselves as crucial mediators in CRC pathophysiology, orchestrating fundamental processes including angiogenesis, immune evasion, metastasis dissemination, and therapeutic resistance. This section elucidates the dual functional capacity of EVs and their molecular cargo, serving simultaneously as: diagnostic and prognostic biomarkers; targeted therapeutic delivery systems in CRC management (**Figure 2**).

## 5 Potential of novel EV-derived biomarkers in colorectal cancer for diagnosis, drug resistance mediation, and treatment

### 5.1 The potential of EV-derived miRNAs as drug carriers for CRC

Building upon the established roles of EV-miRNAs as diagnostic biomarkers (Section 4.1.1) and their regulatory functions in tumour progression (Section 4.1.2), the enhanced stability of exosomal bilayer membrane structures (Section 2.3) enables targeted therapeutic delivery. Specifically, this structural robustness: (1) Protects encapsulated cargo (e.g., miRNAs) from enzymatic degradation during circulation, (2) Facilitates precise delivery to tumour sites through membrane integrity maintenance. For example, leveraging this stability, EV-encapsulated miR-1915-3p enhances chemosensitivity in oxaliplatin-resistant CRC cells by ensuring intact miRNA delivery to resistant cells (126). Similarly, EV-mediated co-delivery of miR-21 and 5-FU capitalizes on membrane stability to synchronize chemotherapeutic agent and gene regulator delivery, effectively overcoming 5-FU resistance in CRC (127).

### 5.2 Emerging roles of EV-derived circRNAs in CRC: dual utility as diagnostic markers and therapeutic targets

According to the content of Section 4.2.2, circular RNAs (circRNAs) have emerged as key regulators in CRC pathogenesis, demonstrating dual utility as both diagnostic and prognostic biomarkers. Notably, hsa_circ_0004771 shows elevated expression in serum samples from CRC patients, with levels decreasing significantly following surgical resection, highlighting its potential as a diagnostic marker (128). Beyond their biomarker potential, circRNAs actively contribute to tumour progression through exosomal pathways. CircSKA3 (hsa_circ_0000467), for instance, drives epithelial-mesenchymal transition (EMT) and metastasis by stabilising the SLUG protein (129). Furthermore, specific circRNAs such as hsa_circ_0004085, which shows upregulation in CRC patients with Fusobacterium nucleatum infection, can be effectively encapsulated within synthetic exosome-like vesicles. This innovative delivery system presents a promising strategy for targeted therapy and overcoming chemoresistance in CRC (130).

### 5.3 EV-derived lncRNAs in CRC: dual-role biomarkers bridging diagnosis and therapeutic resistance

According to the content of Section 4.3.2, long non-coding RNAs (lncRNAs) enriched in extracellular vesicles (EVs) from CRC patients have emerged as clinically valuable biomarkers. A prominent example is lncRNA XIST, which shows significant upregulation in serum EVs of CRC patients and demonstrates positive correlation with established CRC biomarkers including CEA (r = 0.806), CA242 (r = 0.627), CA199 (r = 0.254) and CA153 (r = 0.706) (all P < 0.05). These characteristics position XIST as a promising dual-purpose biomarker for both diagnosis and prognosis (131). Beyond diagnostic applications, EV-associated lncRNAs play a critical role in mediating therapeutic resistance. Notably, elevated levels of EV-derived lncRNA UCA1 correlate with cetuximab resistance in CRC patients. This transfer mechanism underscores UCA1’s potential as a predictive biomarker for treatment response and resistance development. However, the molecular mechanisms underlying these phenomena remain to be further elucidated (132).

### 5.4 EV-associated mRNAs: promising non-invasive diagnostic biomarkers for CRC

Messenger RNAs (mRNAs) encapsulated within EVs demonstrate significant diagnostic potential for CRC. A panel of eight EV-associated mRNA biomarkers—MYC, VEGF, CDX2, CK19, EpCAM, CEA, CD133, and CD24—that show markedly elevated expression in CRC-derived EVs. Among these, VEGF and CD133 exhibit exceptional diagnostic performance, demonstrating both 100% sensitivity and 93% accuracy, thereby representing robust non-invasive biomarkers for CRC detection (133).

### 5.5 EV-derived proteins: promising diagnostic and prognostic biomarkers for CRC

According to the content of Section 4.4.2, proteins cargo within plasma-derived EVs has emerged as a valuable source of diagnostic and prognostic biomarkers for CRC. Notably, increased expression of ORM1 (α-1 acid glycoprotein 1) in plasma EVs demonstrates significant correlation with reduced overall survival, establishing its clinical utility as a prognostic marker (134). Other proteins, including HSPG2, TUBA4A, ITGB3, and TLN1, demonstrate altered expression patterns in CRC patients, indicating their potential utility as prognostic biomarkers (135).

### 5.6 EV cytokines: promising target and marker for CRC

Cytokines encapsulated within tumour-derived EVs, including tumour necrosis factor-α (TNF-α), play critical roles in CRC progression. TNF-α facilitates CRC development by targeting SNAP23, which subsequently enhances TNF-α secretion in EVs. This TNF-α/SNAP23 signalling axis represents both a potential therapeutic target and diagnostic marker for CRC (136).

## 6 Emerging EV-based biomarkers and future directions

In addition, emerging biomarkers including circulating tumour DNA (ctDNA), EV-associated nucleic acids, gut microbial alterations and specific protein markers show promising diagnostic and monitoring potential in CRC (137). EV-based biomarkers provide distinct advantages over conventional biomarkers such as carcinoembryonic antigen (CEA) and circulating microRNAs, including non-invasive sampling, capacity for real-time monitoring, and superior stability and specificity (138). Nevertheless, while accumulating evidence supports the clinical utility of EV-derived molecules in CRC, few clinical trials have systematically assessed their diagnostic performance. Comprehensive clinical validation remains imperative to establish their reliability and effectiveness in routine clinical practice. In summary, EVs and their molecular constituents constitute a robust platform for CRC diagnosis, prognosis and therapeutic development.

## 7 Conclusion and perspectives

Colorectal cancer (CRC) demonstrates increasing global prevalence, particularly among younger demographic groups, highlighting the pressing requirement for improved early detection and therapeutic approaches. The frequently non-specific early clinical manifestations of CRC commonly result in diagnosis at advanced disease stages, substantially diminishing patient prognosis and survival outcomes. This clinical challenge has established the discovery of novel biomarkers for early detection and intervention as a paramount research priority in CRC management.

Extracellular vesicles (EVs) are detectable in diverse biological fluids and encapsulate numerous biomolecules, including proteins and nucleic acids. These EV-associated components play pivotal role in CRC pathogenesis through regulation of critical processes including angiogenesis, cellular proliferation and migration activity. Such functional properties render EV-derived biomolecules particularly promising candidates for CRC diagnosis and prognostic assessment. Among various sample types, plasma-derived EVs have attracted the most extensive research focus due to: the non-invasive nature of plasma collection; their inherent stability and abundant; their direct relevance to CRC pathology. Nevertheless, plasma presents technical challenges for EV isolation and analysis, primarily due to interference from soluble proteins and aggregates, which may compromise the precision of biomarker detection.

Current methodologies for detecting microRNAs (miRNAs) in exosomes derived from serum, plasma and cell lines primarily employ quantitative reverse transcription polymerase chain reaction (qRT-PCR). Similarly, identification of trace RNA in exosomes f samples from these sources predominantly relies on qRT-PCR. However, the low abundance of these nucleic acids in exosomes frequently restricts the sensitivity of conventional detection approaches. To overcome these limitations, digital PCR (dPCR) has emerged as an increasingly utilised technique offering exceptional sensitivity for nucleic acid quantification at single-molecule resolution. Through sample partitioning into numerous microscopic reaction chambers, dPCR facilitates precise nucleic acids quantification in exosomes, thereby addressing the constraints of traditional PCR for low-abundance targets. Although dPCR has not yet achieved widespread adoption in exosome research, its capacity to improve nucleic acid detection accuracy positions it as a promising methodology for advancing EV-based diagnostic applications.

Despite significant progress in EV isolation methodologies, persistent challenges remain in efficiently isolating and characterisation tumour-derived EVs. Establishing standardised protocols and robust quality control measures represents a critical requirement to enhance yield, purity and reproducibility in EV research. Such standardisation will accelerate the clinical translation of EV-based diagnostic and therapeutic approaches, ultimately improving the effectiveness of CRC detection and treatment strategies.

In summary, EVs represent constitute a promising for CRC biomarker discovery, providing valuable insights into tumour biology and revealing potential therapeutic targets. The implementation of advanced technologies such as dPCR, coupled with the development of standardised isolation protocols, will prove essential for addressing current limitations and realising the complete potential of EVs in CRC diagnostics and personalised medicine. Future investigations should prioritise: (1) validation of EV-derived biomarkers in large-scale clinical cohorts, and (2) exploration of their clinical utility in guiding therapeutic decision-making—ultimately enhancing outcomes for CRC patients.
